# Whodunnit? Electrophysiological Correlates of Agency Judgements

**DOI:** 10.1371/journal.pone.0028657

**Published:** 2011-12-14

**Authors:** Simone Kühn, Ivan Nenchev, Patrick Haggard, Marcel Brass, Jürgen Gallinat, Martin Voss

**Affiliations:** 1 Department of Experimental Psychology, Faculty of Psychology and Educational Sciences, and Ghent Institute for Functional and Metabolic Imaging, Ghent University, Ghent, Belgium; 2 Institute of Cognitive Neuroscience, Department of Psychology, University College London, London, United Kingdom; 3 Department of Psychiatry and Psychotherapy, St Hedwig Krankenhaus, Charité University Medicine, Berlin, Germany; Royal Holloway, University of London, United Kingdom of America

## Abstract

Sense of agency refers to the feeling that “I” am responsible for those external events that are directly produced by one's own voluntary actions. Recent theories distinguish between a non-conceptual “feeling” of agency linked to changes in the processing of self-generated sensory events, and a higher-order judgement of agency, which attributes sensory events to the self. In the current study we explore the neural correlates of the judgement of agency by means of electrophysiology. We measured event-related potentials to tones that were either perceived or not perceived as triggered by participants' voluntary actions and related these potentials to later judgements of agency over the tones. Replicating earlier findings on predictive sensory attenuation, we found that the N1 component was attenuated for congruent tones that corresponded to the learned action-effect mapping as opposed to incongruent tones that did not correspond to the previously acquired associations between actions and tones. The P3a component, but not the N1, directly reflected the judgement of agency: deflections in this component were greater for tones judged as self-generated than for tones judged as externally produced. The fact that the outcome of the later agency judgement was predictable based on the P3a component demonstrates that agency judgements incorporate early information processing components and are not purely reconstructive, post-hoc evaluations generated at time of judgement.

## Introduction

Humans possess a sense of agency, which is a central aspect of voluntary action. Sense of agency refers to the experience that one is the agent of one's own actions. This provides us with the feeling of control, and may also be the basis of our wider understanding of physical causation (Maine de Biran quoted in [1], p. 11). In addition, sense of agency accompanies the human capacity to detect and distinguish whether sensory signals are the result of self-generated actions or other environmental events [2]. Recently, a two-step account of agency has been proposed. In the first step, different agency indicators lead to a feeling of agency. In a second step this feeling of agency is then processed by conceptual modules to make judgements of attribution [3], [4]. One might speculate about the relations between feeling of agency and judgement of agency. Both could be based on independent processes in different brain regions that do not interact with one another, e.g. feeling of agency might be based on brain regions involved in basic motor or auditory processes whereas the judgement of agency might be based on brain regions such as parietal association cortex [5]. If both processes were independent one would predict no relation between feeling of agency and judgement of agency. One would then call the outcome of the later judgement a confabulation or post-hoc reconstruction. On the other hand the judgement of agency could depend on sensorimotor signals generated at the time of agency that is also relevant for the feeling of agency. Attributional judgements of agency would then make use of information from those lower level feelings of agency.

Interestingly, to our knowledge no study has directly investigated whether sensory processing involved in the feeling of agency could form the basis of judgements of agency. However, it has been proposed that internal forward models may provide an internal prediction that can be used to distinguish between self- and externally-generated sensory events [6]–[8], and thus establish agency. Forward models use an “efference copy” of the motor command to predict the consequences of actions, thus increasing the salience of sensations with an external cause relative to self-generated sensations [9]. Such sensory attenuation has been found in the sensorimotor domain (e.g. [10], [11]) as well as in the auditory domain (e.g. [12]). An electrophysiological marker of sensory attenuation in the auditory domain is the N1 component [12]–[14]. Another prominent but slightly later electrophysiological marker that is sensitive to the detection of unexpected, “odd” or target events is the P3 component that occurs around 300 ms after tone onset [15]–[17]. Since participants knew in advance that they would have to judge who caused the tone, tones that feel “odd” or slightly unexpected could be the ones that are later attributed to somebody else.

It is common to discriminate between a P3a and a P3b [18]. A parietocentral P3b is elicited by task-relevant deviant stimuli that are attended to, such as response targets, whereas deviant stimuli that are odd or more salient elicit a slightly earlier positive deflection that has a frontocentral scalp topography the so-called P3a. The P3a has been interpreted as neural correlate of the orienting response [19], We did not expect changes in the range of the P3b because every tone was a target in the sense that participants had to judge them and the P3b is usually observed when only a few responses are required and a whole train of stimuli is presented.

In contrast to the modality-specific N1 that is usually classified as an “exogenous” unconscious ERP component, the P3a has been implicated in event detection processes in and is considered as a so-called “endogenous” and component associated with conscious attention processes [17]. We consider both, the N1 as well as the P3 as markers of early agency processing that underly the feeling of agency.

We set out to investigate whether or not explicit judgements of agency, that are assessed considerably later in time than the action and its effects in the environment, are based on information processing at the time of the actual event. Specifically, we explored N1 and P3a potentials to action-effect tones, as potential electrophysiological markers of first-step feeling of agency processing. N1 reflecting unconscious sensorimotor processes and P3a reflecting early conscious stimulus processes reflecting expectancies and oddness. If the physiological markers predict the outcome of the later agency judgement, this suggests that agency judgements draw on information of lower level feelings of agency. To explore this question, we used a task in which participants were asked to press keys that elicit effects in the environment [2]. First participants learned a mapping between right and left button presses and the associated high or low tone that their actions elicited. Then they were told that in the following phase of the experiment the tones they hear could either be the result of their own button press, or produced by one of the experimenters who was performing the same task in parallel. Actually the tones were always randomly generated by the computer and presented after the button press of the participant. After each tone, participants had to provide a judgement of agency, by rating on a visual analogue scale the extent to which they felt that they or the experimenter produced the tone. The tones were either congruent or incongruent to the previously learned association, and were presented with different temporal delays to evoke uncertainty over who caused the outcome.

To demonstrate the classical sensory attenuation effect and to provide evidence that the action to tone mapping has been learned in the training phases we planned to compare tones that were congruent and incongruent to the associated mapping. To investigate the extent to which agency judgements incorporate early information processing components as reflected in the tone-elicited N1 and the P3a we divided trials according to whether the subsequent judgement indicated a self- or externally-generated tone. If explicit judgements of agency are mere retrospective confabulations at the time of judgement, unrelated to tone processing, then auditory evoked potentials should be identical in self- and externally-generated conditions. In contrast, a predictive account of agency would assume that ERPs are attenuated for tones that are subsequently explicitly judged to be self-generated.

## Methods

### Participants

Seventeen participants (age range: 22 to 51 years, mean 33.5 years, 9 female, 8 male) participated in the experiment and gave written informed consent. The study was approved by the ethics committee at Charité University Hospital and conducted according to the Declaration of Helsinki. All participants had normal or corrected-to-normal vision. All the participants were neurologically and psychiatrically healthy. Participants were paid for their participation.

### Procedure

**Figure 1:**
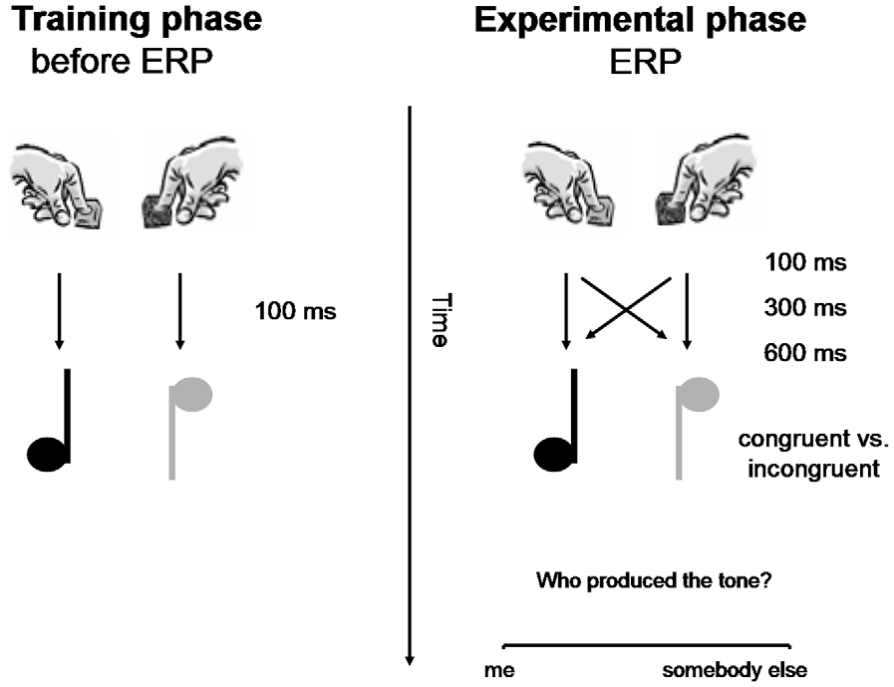
Schematic Drawing of the experimental design.

The participants were seated in a comfortable armchair in a dim sound-attenuated room. Visual stimuli were presented on a computer screen and auditory stimuli via headphones. The experimental design was based on Sato and Yasuda [2]. At the beginning of the experiment, participants performed 300 training trials to learn the relationship between actions and their consequences (*Figure 1*). Participants were instructed to press the left button (left *Alt* key on a key board) with the left index finger and the right button (right *Alt* key) with the right index finger in random order whenever a white square was presented for 200 ms on the screen. After each button press, a 400 Hz or a 800 Hz tone was presented after 100 ms for 200 ms. The inter-trial interval varied between 2000, 2500 and 3000 ms duration. The assignment of buttons to tones was consistent for each participant, but counterbalanced across participants. Then the EEG cap was put on and the actual experiment started. Participants were instructed to freely choose between pressing the right or left button whenever a white square was presented on the screen (as in the learning phase). They were told that tones could be either produced by themselves (as in the learning phase) or by the experimenter who was seated in front of a computer behind a folding screen. In reality, the experimenter did not produce any action–effects, but all tones were generated by the computer and presented with a specific delay after the button press of the participant. In the *congruent tone condition*, each button press evoked the same tone that had followed right and left button press in the learning session. By contrast, in the *incongruent tone condition*, a tone that differed from the predicted tone followed the button press. Moreover, the onset of the tone was manipulated and varied between 100 ms, 300 ms and 600 ms. Conguency and delay was manipulated to evoke uncertainty about self-agency. After each trial and a delay of 3000 ms, participants had to judge on a visual analogue rating scale by means of a computer mouse, if they produced the action–effect or the experimenter did (“Who produced the tone?”,1 = “Me”, 100 = “Somebody else”). Each of the six experimental blocks consisted of 60 trials. After each even experimental block, a short period of the training phase (20 trials) was repeated to refresh the action-effect mapping. After each block participants were allowed a short break.

### Electrophysiological Recordings

Brainwaves were measured with 31 electrodes mounted in an elastic electrode cap (EASYCAP) according to a modified 10–20 setting (with the electrodes Fp1, Fp2, F7, F3, Fz, F4, F8, FC5, FC6, FC1, FC2, T7, C3, Cz, FCz, C4, T8, TP9, CP5, CP1, CP2 CP6, TP10, P7, P3, Pz, P4, P8, O1, Oz, O2). EEG signals were referenced to FCz. Impedances of the electrodes were kept below 4 KΩ. The electrooculogram (EOG) was recorded with bipolar montage. The vertical EOG was measured with two electrodes placed above and below the left eye. The signal was re-referenced offline to the average signal of the electrodes placed on the left and right mastoid. The sampling rate was 512 Hz for all electrodes. The continuous EEG was filtered off-line with a band pass filter of 0.1–30 Hz.

### Data Analysis

#### ERP

EEG data were analyzed using BrainVision Analyzer (MES, Munich). ERPs were time-locked to the onset of the tone with a time-window (segmentation) from −200 to 1000 ms. Baseline correction was performed for the time frame of −200 to 0 ms. Segments were removed from the analysis if the standard deviation of any scalp electrode exceeded ±20 µV within a sliding time window of 200 ms or if the standard deviation of the EOG within the same time window exceeded ±40 µV. The remaining artifact free segments were averaged separately for the different conditions (see below).

Our analysis first focused on differences between congruent and incongruent tones across all trials, including three different tone onsets (100 ms, 300 ms and 600 ms).

Secondly, in order to assess tone-related differences based on the outcome of the agency judgements, we divided all trials with congruent tones presented with a medium delay (300 ms) into those rated as *self-produced* vs. *experimenter-produced* according to a median split on the agency scale. This analysis focuses on congruent tones presented medium delay, because the uncertainty of agency is considerably high and error processing based on incongruency of tone identity can be excluded.

The amplitude of the N1 was determined in the averaged segments as the mean within the time window of 110 to 130 ms following tone onset. The amplitude of the P3a was determined mean within the time window of 370–390 ms post stimulus and expressed as µV.

Our main interest was on the N1 and P3a in a fronto-central ROI (comprising electrodes F3, Fz, F4, FC1, FCz, FC2 – measured as a mean amplitude across these electrodes [13], [20]). Based on our a priori hypotheses that incongruent tones should elicit a bigger N1 than congruent tones and the hypothesis that tones ascribed to the experimenter should elicit an enhanced amplitude in N1 or P3a compared to self-ascribed tones, we conducted two repeated measures ANOVAs: (1) One comprising the factors component (N1 vs. P3a) and condition (congruent vs. incongruent tones); (2) the other comprising the factors component (N1 vs. P3a) and condition (tones attributed to “me” vs. tones attributed to “somebody else”). The assignment of trials to the condition “me” or “somebody else” were confined to trials with congruent tones and of medium delay (300 ms) because this condition involved most ambiguity and were defined by a median split of all agency judgements in this condition of each participant.

## Results

### Behavioral Results

**Figure 2:**
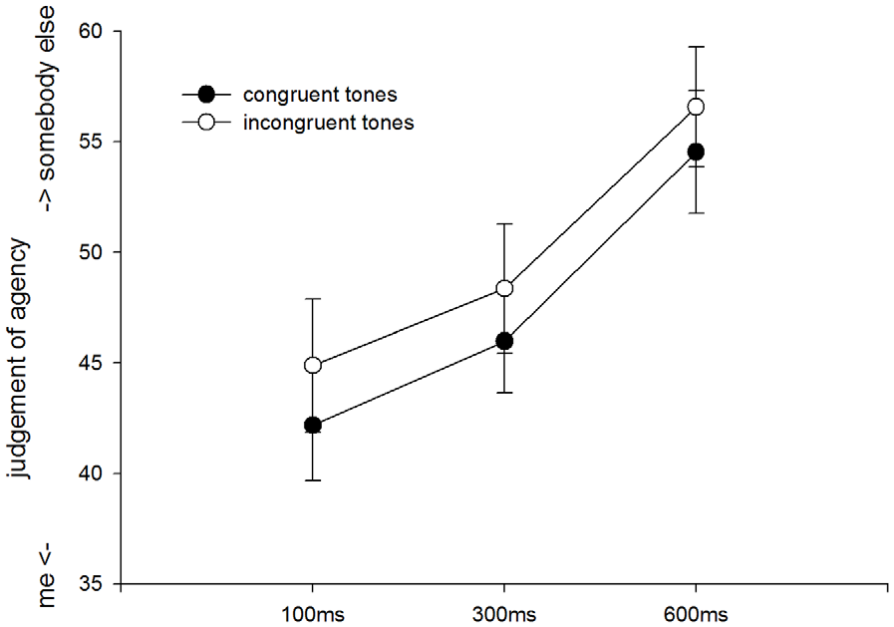
Behavioral effects of agency judgement. Error bars depict standard error of the mean.

The rating scores on the agency scale were analyzed using a repeated measures analysis of variance (ANOVA) with the factor tone congruency (congruent vs. incongruent) and delay (100, 300, 600 ms). This analysis revealed a main effect of tone congruency (*F*(1,16) = 5.65, *p*<0.05) and a main effect of delay (*F*(2,32) = 10.34, *p*<0.001) (*Figure 2*). No interaction between tone congruency and delay was found (*F*(2,32) = .15, *p* = .86). The main effect of tone congruency was based on stronger self-agency (“me”) judgements when actual and predicted tones were congruent compared to incongruent. The main effect of delay was based on a reduction of self-agency (“me”) judgements with increasing delays.

### ERP Results

**Figure 3:**
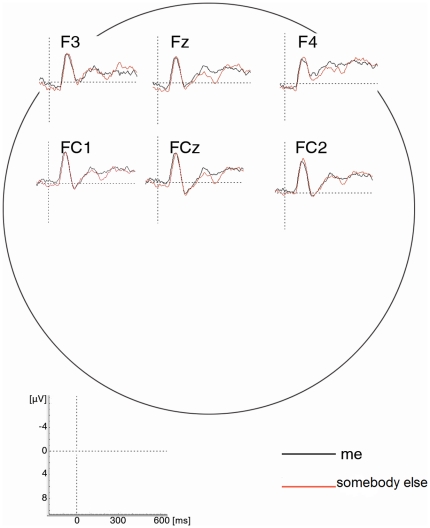
Tone-locked ERPs of “me” vs. “somebody else” agency judgement on frontal electrodes (in congruent condition with medium delay).

**Figure 4:**
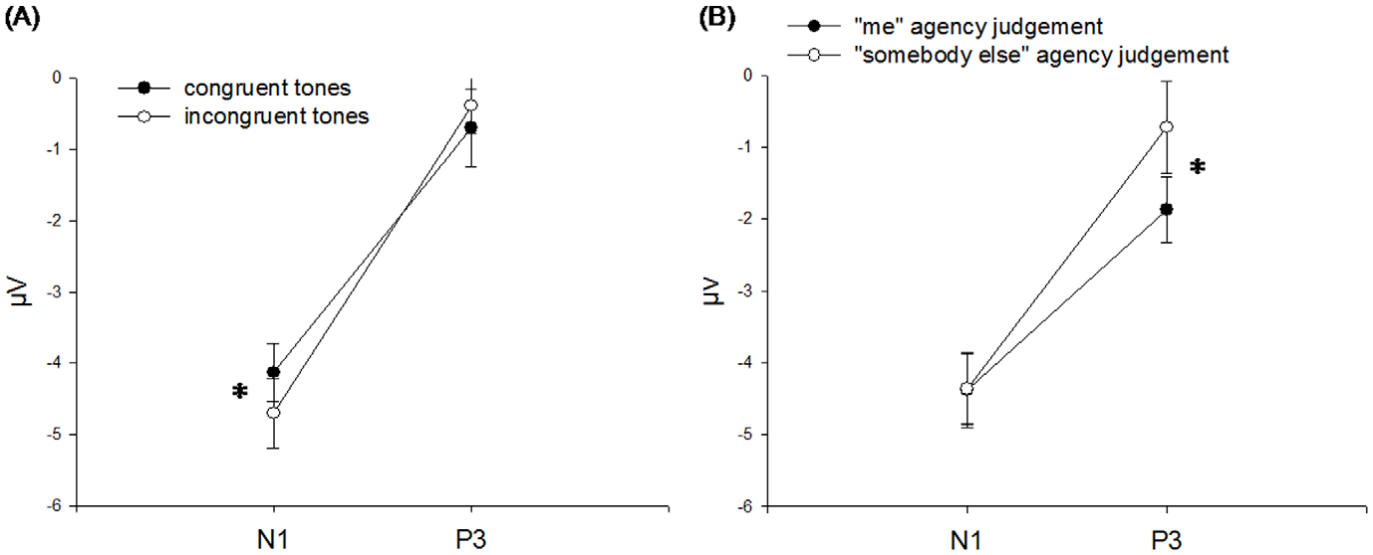
Plots displaying mean signal averaged over electrodes F3, Fz, F4, FC1, FCz, FC2. (A) Interaction plot of component (N1 vs. P3a) and condition (congruent vs. incongruent tones), (B) Interaction plot of component (N1 vs. P3a) and condition (“me” vs. ”somebody else” agency judgement, median split in trials with congruent tones and delay 300 ms). * indicates a significant post-hoc t-test.

The tone-locked ERPs are plotted in *Figure 3*. The repeated measures ANOVA comparing N1 and P3a amplitudes in response to congruent and incongruent tone condition across all delays revealed a main effect of component (*F*(1,16) = 94.46, *p*<0.001) and a significant interaction of the factors component and condition (*F*(1,16) = 5.87, *p*<0.05) indicating a more pronounced difference between congruent and incongruent tones for the N1 but not in the P3a component (*Figure 4A*). Post-hoc t-tests revealed a significant difference between conditions in the N1 (*t*(16) = 2.32, *p*<0.05) not in the P3a (*t*(16) = −0.92, *p* = 0.37). The presence of sensory attenuation in the congruent compared to incongruent tone condition shows that participants have learned the association between button presses and resulting tones.

In contrast to this a similar analysis based on the judgements revealed a main effect of component (*F*(1,16) = 84.54, *p*<0.001) and a significant interaction of the factors component and condition (*F*(1,16) = 5.45, *p*<0.05) indicating a more pronounced difference between “me” and “somebody else” judgements for the P3a but not for the N1 amplitude (*Figure 4B*). Post-hoc t-tests revealed a significant difference between conditions for P3a amplitude with the “me” ERPs being attenuated compared to the “somebody else” judgements (*t*(16) = −2.13, *p*<0.05) but not in the N1 amplitude (*t*(16) = −0.03, *p* = 0.98). This finding indicates that the judgement of agency does not draw on early sensory attenuation processes in the range of the N1 but onto later possibly more cognitive mechanisms reflected in the P3a.

## Discussion

The current study set out to test whether differential electrophysiological responses in response to events predict later explicit agency judgements concerning these events. More specific, we aimed at exploring whether agency judgements are related to processes that take place immediately after the effect in the environment has happened or whether agency judgements are retrospectively constructed once the judgement has to be made. To ensure that the action to tone mapping has been learned in the training phases, we compared tones that corresponded to the learned association between button presses and action-effects (congruent) with tones that did not correspond to the learned association (incongruent). In line with our hypothesis we found sensory attenuation, namely a reduction of the N1 component for congruent compared to incongruent tones, demonstrating that the association of actions and action consequences has been learned. The N1 component is elicited by auditory stimuli and assumed to be generated in auditory cortex [21], [22]. It has been shown that if participants expect self-initiated auditory stimuli that are presented after a fixed and therefore predictable delay the N1 component is reduced compared to randomly presented tones [12]–[14]. Similarly visual N1 attenuation has been shown when subjects compared to a computer generated the visual action effect [23].

To explore the extent to which agency judgements incorporate early information processing steps as reflected in the tone-elicited N1 and the slightly later P3a, we divided identical congruent trials presented with the medium tone delay according to judgements of whether each individual tone was self- or externally-produced. If feeling of agency and judgement of agency redraw on different information and the judgement is solely based on reconstructive confabulation at time of judgement one would predict no systematic differences in ERPs elicited by the tone presentation, whereas an account that assumes information flow from feeling of agency to judgement of agency would predict an attenuation of components whenever agency is attributed to the self. In line with the latter account we found a stronger P3a component for tones that were judged to be generated by somebody else (namely the experimenter) compared to those judged to be generated by oneself. The P3a is a prominent electrophysiological marker that is sensitive to attention processes [15] it has been shown to be augmented whenever unexpected or “odd” stimuli are presented, while expected stimuli lead to an attenuated response (for an overview [17]). Previous EEG studies have located the generators of the P3 in the temporal or parietal lobe [24]–[26]. The observed association between judgement of agency and the P3a is therefore in line with a multitude of fMRI studies associating agency processing with the temporo-parietal junction [5], [27], [28].

It has been shown previously that a P3a component can be elicited without a concurrent N1 increase ([17]; dissociation of MMN and P3a [29]) arguing against a strongly coupled chain of auditory processing starting with N1 that is followed by a P3a. The presence of an association between P3a and later agency judgement in the absence of an N1 effect is in line with the notion that these processing stages of N1 and P3a can at least partly run independently. From the present data we cannot derive *why* the participants ERPs sometimes seem to signal that an identical tone is “odd” or unexpected, but we observed that the occurence of an enhanced P3a predicts the attribution of the tone to the experimenter. We speculate that the differences in the range of the P3a after the physically identical congruent trials (tone delay 300 ms) are based on fluctuations of attention: on trials in which participants are slightly distracted or engaged in mind wandering, tones might have a higher probability of being perceived as unexpected or “odd”. Further research is needed in order to explore the underlying psychological processes of the reduced P3a that predicts the later self-agency judgement.

To summarize, the aim of the present study was to explore the neural correlates of explicit agency judgements. Participants learned that certain actions resulted in certain consequences in the environment (tones). They were then introduced to an ambiguous context in which they had to judge whether presented tones where self-generated or externally produced. A comparison of congruent and incongruent tones (with respect to the previously learned associations) revealed a reduction of the N1 component for congruent tones, showing that participants learned the mapping and thus attenuated expected action effects. The outcome of a later agency judgement was predictable based on a P3a component demonstrating that agency judgements incorporate early information processing components within the range of the evoked potential and are not purely reconstructive post-hoc evaluations generated at the time of judgement.

However, the agency judgements were not based on exogeneous, unconsious sensorimotor processes as reflected in the N1, but on later endogeneous processes within the range of the P3a possibly reflecting the detection of the tones' unexpectedness or oddness.
